# Using Digital Speech Assessments to Detect Early Signs of Cognitive Impairment

**DOI:** 10.3389/fdgth.2021.749758

**Published:** 2021-10-27

**Authors:** Jessica Robin, Mengdan Xu, Liam D. Kaufman, William Simpson

**Affiliations:** ^1^Winterlight Labs, Toronto, ON, Canada; ^2^Department of Psychiatry and Behavioural Neuroscience, McMaster University, Hamilton, ON, Canada

**Keywords:** speech, digital biomarker, language, mild cognitive impairment, Alzheimer's disease

## Abstract

Detecting early signs of cognitive decline is crucial for early detection and treatment of Alzheimer's Disease. Most of the current screening tools for Alzheimer's Disease represent a significant burden, requiring invasive procedures, or intensive and costly clinical testing. Recent findings have highlighted changes to speech and language patterns that occur in Alzheimer's Disease, and may be detectable prior to diagnosis. Automated tools to assess speech have been developed that can be used on a smartphone or tablet, from one's home, in under 10 min. In this study, we present the results of a study of older adults who completed a digital speech assessment task over a 6-month period. Participants were grouped according to those who scored above (*N* = 18) or below (*N* = 18) the recommended threshold for detecting cognitive impairment on the Montreal Cognitive Assessment (MoCA) and those with diagnoses of mild cognitive impairment (MCI) or early Alzheimer's Disease (AD) (*N* = 14). Older adults who scored above the MoCA threshold had better performance on speech composites reflecting language coherence, information richness, syntactic complexity, and word finding abilities. Those with MCI and AD showed more rapid decline in the coherence of language from baseline to 6-month follow-up, suggesting that this score may be useful both for detecting cognitive decline and monitoring change over time. This study demonstrates that automated speech assessments have potential as sensitive tools to detect early signs of cognitive impairment and monitor progression over time.

## Introduction

There is a clear and pressing need for sensitive tools to aid in the detection and monitoring of mild cognitive impairment (MCI) and Alzheimer's disease (AD) (1–5). Given the prevalence of Alzheimer's disease and the aging populations in many countries (6), it will be essential to have tools to help identify the presence of cognitive impairment relating to MCI and AD that can be deployed frequently, and at scale. This need will only increase as effective interventions are developed, requiring the ability to identify patients early in order to facilitate prevention or treatment of disease (7). With recent shifts toward telemedicine and increased digital literacy of the aging population, digital health tools are ideally poised to meet the needs for novel solutions. Digital assessments that can be accessed on a smartphone or tablet, completed from home and periodically repeated, would vastly improve the accessibility of AD screening compared to current clinical standards that require clinical visits, extensive neuropsychological testing or invasive procedures.

Speech is a promising modality for digital assessments of cognitive abilities. Producing speech samples is a highly ecologically valid task, instrumental to daily functioning and requiring little instruction. The pervasiveness of high-quality microphones in smart devices makes the recording of speech samples straightforward, not requiring additional equipment or sensors. Advances in signal processing and natural language processing have enabled objective analysis of speech samples for both their acoustic properties and linguistic content, providing a window into motor, linguistic and cognitive abilities. Most importantly, previous research has extensively shown that speech patterns are affected in MCI and AD, demonstrating the clinical relevance of speech for detecting cognitive impairment and dementia (8–12).

Much of the previous work studying speech patterns in AD using automated speech analysis has focused on multivariate classification models to differentiate those with AD or MCI from healthy controls based on their speech (9–11). These models generally achieve high accuracy (80–90%) demonstrating that there are robust differences between clinical groups based on speech characteristics (13–20). While classification models are well-suited to potentially aid with disease diagnosis or screening, they can be challenging to interpret based on their multivariate nature which may make it difficult to determine which aspects of speech and language contribute to the classification. Furthermore, such models are not ideal for longitudinal tracking to determine if symptoms are worsening over time or improving with treatment. A smaller set of studies have examined longitudinal changes in speech patterns, suggesting that notable changes to linguistic features may occur as an early sign of incipient AD, but mostly rely on manual coding or subjective ratings of speech, and are limited to small or specialized samples (21–25). One recent study of a large community-based cohort found promising results indicating that linguistic characteristics such as repetition and errors can predict later AD diagnosis, but focused on written text analysis instead of speech (26).

In the present study, we collected longitudinal speech assessments from a community-recruited sample of older adults and individuals with MCI and early AD recruited at a clinical site. Speech was recorded using a tablet-based digital speech assessment including a range of structured and unstructured speech tasks. We analyzed speech characteristics reflecting different domains of speech and language abilities, to obtain interpretable measures of speech and language function suitable for repeated testing. We tested how these measures differed between groups and related to other measures of cognitive function to assess their potential as indicators of cognitive impairment relating to MCI and AD. Finally, we compared longitudinal performance based on 6-month follow-up assessments to determine how digital speech assessments can be used to track changes over time. Together, the results from this study serve as a proof-of-concept of how digital speech assessments can be used as quick, naturalistic, remote assessments of language abilities and cognitive status.

## Methods

### Participants

Participants were recruited from the community and at clinical sites to participate in a longitudinal speech assessment study. For community participants, eligibility criteria included being between the ages of 50 and 95, being a fluent speaker of English, and having no diagnosis of dementia, memory impairment, recent concussion or traumatic brain injury, or uncorrected hearing or visual impairment. Participants recruited at clinical sites had diagnoses of MCI or AD confirmed by the principal investigator at the site (27, 28). Eligibility criteria were otherwise the same as the normative sample, with the additional exclusion criteria of global scores >1 on the Clinical Dementia Rating (CDR) scale or a concurrent diagnosis of: depression, anxiety, schizophrenia, bipolar disorder, or alcohol/substance use disorders. All participants provided informed consent to participate in the study, and the study protocols were approved by an independent research ethics institutional review board (Advarra).

### Study Procedure

Participants completed three study visits, at Baseline, and 1- and 6-month follow ups. At each visit, participants completed a standardized speech assessment on an iPad, facilitated by a trained administrator. In addition to the speech assessment, at Baseline and 6-month visits, participants completed neuropsychological assessments, including the Montreal Cognitive Assessment [MoCA; (29)] and the Alzheimer's Disease Assessment Scale–Cognitive Subscale [ADAS-Cog; (30)]. The MCI/AD group additionally completed other neuropsychological assessments, including the Symbol Digit Modalities Test [SDMT; (31)], the Hopkins Verbal Learning Test [HVLT; (32)], Judgement of Line Orientation test [JLO; (33)], Digit Span forward and backward (34), and Trails A and B (35).

#### Speech Assessment

The Winterlight Assessment (WLA) was developed to record and analyze speech *via* a smartphone/tablet app, and has been used in previous normative studies and clinical trials (36–38). The WLA consists of a series of speech tasks, in which participants are prompted to produce speech and are recorded through the device's microphone. The speech tasks included in the WLA are based on standard neuropsychological speech and language assessments. In the present study, the WLA included six speech tasks, and took an average of 5–10 min to complete.

**Figure 1:**
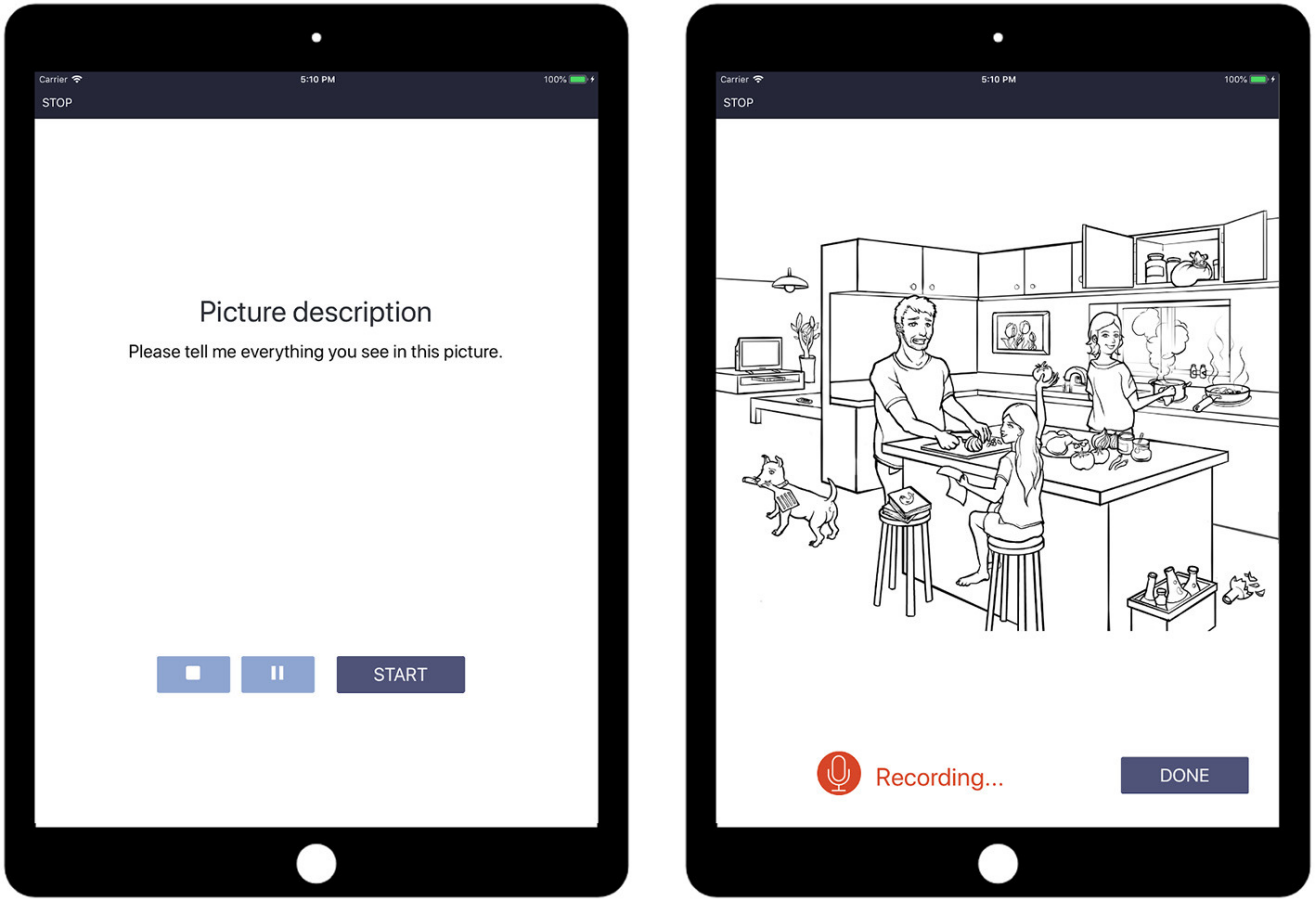
Schematic of the picture description task, part of the Winterlight Assessment (WLA).

Speech tasks included picture description, paragraph reading and recall, letter and semantic fluency, and object naming. For the present paper, we focus on the picture description task, which most closely approximates spontaneous speech and has been commonly used in previous research on MCI and AD (39–42). In the task, the instruction, “Please tell me everything you see in this picture” is presented visually and auditorily and then a static line drawing of a scene is displayed on the screen (Figure 1). Participants describe the image in their own words, with no time limit, and are recorded. Tasks of this type have been shown to be good proxies for spontaneous discourse and are used in standard aphasia assessments (41, 43). The WLA includes six unique and proprietary images developed to match the Cookie Theft picture [from the Boston Diagnostic Aphasia Examination, Goodglass et al. (44)] in lexico-syntactic complexity and the amount of information units. At each assessment, participants completed two picture description tasks, and performance was averaged across the two.

### Data Analysis

#### Speech Analysis

Speech recordings were analyzed using the Winterlight Labs speech analysis platform. The speech recordings made by the participant were transcribed, and linguistic and acoustic variables were extracted through automated speech analysis. The generation of annotations such as speaker segmentation, transcription, and utterance segmentation was performed by trained raters using a secure, cloud-based web application. Any recordings that didn't contain participant speech or that had major audio quality issues were removed from analyses. Data processing and feature extraction were performed using the Winterlight Labs pipeline (www.winterlightlabs.com) using Python-based standard acoustic and language processing libraries and proprietary custom code. Over 500 speech variables were computed based on each speech recording and its accompanying transcript. Speech variables were either directly computed from the sound file or annotated transcript using standard, open-source signal processing or NLP algorithms [e.g., spaCy and the Stanford parser; (45, 46)], or using custom code to calculate novel variables (e.g., calculating the number of correct information units described by comparing the words in a transcript with a list of items contained in the picture). The set of speech variables reflects the acoustic (e.g., properties of the sound wave, speech rate, number of pauses), lexical (e.g., rates and types of words used, and their characteristics such as frequency and imageability, which reflect how commonly words are used and how easy they are to picture, respectively), semantic (relating to the meaning of the words, e.g., semantic relatedness of subsequent utterances, semantic relatedness of utterances to the items in the picture) and syntactic (relating to the grammar of the sentences, e.g., syntactic complexity, use of different syntactic constructions) aspects of each speech sample.

In order to reduce the number of variables and cluster related variables together, we created eight aggregate scores reflecting different aspects of speech and language abilities. Variables were selected and aggregated based on the domains of language that have been reported to be affected in MCI/AD in previous literature (14, 25, 47–52). Note that while acoustic features have been reported to differ in MCI/AD (14, 18), the clinical interpretability of these features is not as straightforward and many classification models do not report on which features contribute to model performance, so they were not included in the aggregates scores in this study. In order to form the aggregate scores, each variable was standardized based on a normative sample to convert it to a unit-free standard score, and given a positive or negative weighting based on whether higher scores represent better or worse language abilities, and averaged together. The eight aggregate scores are described below, and lists of all component variables are included in Supplementary Material:

**Discourse mapping** measures the repetitiveness or circularity of speech, with higher scores indicating less repetitive speech. Component variables include cosine distance metrics and graph theoretical metrics of discourse.

**Global coherence** measures how related the words produced are to the key items in the picture, with higher scores indicating higher coherence to the themes in the picture. Component variables include cosine distance metrics comparing the semantic similarity of utterances with the key words for each picture stimulus.

**Information units** measure how informative and detailed the picture description is, with higher scores indicating greater detail richness. Component variables include scores for how many of each detail type is mentioned, coded based on each picture stimulus.

**Lexical richness** measures the complexity and diversity of words used, with higher scores indicating a richer, more complex vocabulary. Component variables include the rates of different word types, the characteristics of words used (frequency, familiarity, age of acquisition) and measures of vocabulary richness.

**Local coherence** measures how related successive utterances are to one another, with higher scores indicating that utterances are more related to one another. Component variables include cosine distance metrics comparing the semantic similarity of subsequent utterances.

**Sentiment** measures whether more positive or negative words are used, with higher scores indicating speech with more positive valence. Component variables include average valence scores for nouns, verbs, and all word types.

**Syntactic complexity** measures the complexity of syntactic structures used, with higher scores indicating more complex syntax. Component variables include the measures of the length of phrases, the complexity of clauses, and the rates of different syntactic structures.

**Word finding difficulty** measures the fluidity and fluency of speech, with higher scores indicating more signs of slowed or hesitant speech. Component variables include the rate of speech, duration of words and number, and duration of filled (e.g., um, uh) and unfilled pauses.

#### Statistical Analysis

Statistical analysis was performed using R software and packages including lme4, lmerTest, and tidyverse (53–56). Linear mixed models with random intercepts were used to test for significant effects of group and the interaction between group and visit on the speech aggregate scores, while controlling for demographic variables.

## Results

### Participant Demographics

Seventy-five participants completed a speech assessment at baseline, 1- and 6-month longitudinal follow-up visit. For the purposes of this study, we compared participants in three groups: those that scored ≥26 points on the MoCA (29) at both baseline and 6-month assessments (High MoCA), those that scored <26 points on the MoCA at both assessments (Low MoCA), and those with MCI or AD diagnoses (MCI/AD). Sample size and demographics of each group are shown in Table 1. A chi-squared test showed no difference in the ratio of males to females across groups (χ^2^ = 2.74, *p* = 0.25). ANOVAs testing the effect of group showed that MoCA scores [*F*_(2, 46)_ = 33.89, *p* < 0.001] and mean age [*F*_(2, 47)_ = 7.85, *p* = 0.001] differed across groups, while the mean number of years of education did not differ significantly [*F*_(2, 47)_ = 2.56, *p* = 0.09]. ADAS-Cog data was not available for all participants, but there was a group difference in total scores [*F*_(2, 32)_ = 14.15, *p* < 0.001] in the subset of participants with scores available. Mean values and standard deviations for each group are listed in Table 1. We included factors of sex, age, and years of education in subsequent analyses to control for variation based on demographic factors.

**Table 1:** Baseline demographic information and clinical scores for each of the study groups.

	**High MoCA** **(*n* = 18)**	**Low MoCA** **(*n* = 18)**	**MCI/AD** **(*n* = 14)**
Sex (M/F)	7 M/11 F	4 M/14 F	7 M/7 F
Mean age (SD)	66.2 (11.8)	79.3 (11.5)	76.1 (5.2)
Mean years of education (SD)	15.3 (3.5)	14.1 (2.2)	16.6 (3.3)
Mean MoCA score (SD)	27.5 (1.2)	21.2 (3.2)	19.6 (3.9)
Mean ADAS-Cog score (SD; *n*)	4.5 (3.3; *n* = 14)	11.4 (3.9; *n* = 7)	13.6 (6.0; *n* = 14)

### Speech Differences Based on Cognitive Status

**Figure 2:**
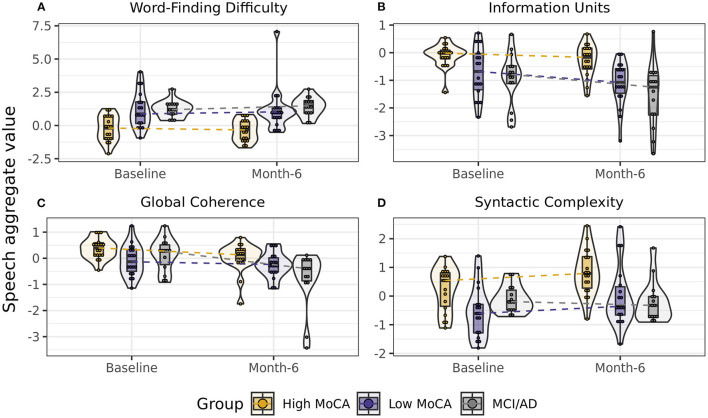
Scores on speech aggregates that showed significant differences between groups. **(A)** Word-finding difficulty scores, reflecting increased pauses, and slower speech, were highest in the MCI/AD group and lowest in the High MoCA group. **(B)** Information units scores, reflecting how much content was accurately described in the pictures, were highest in the High MoCA group and lowest in the MCI/AD group. **(C)** Global coherence scores, reflecting the semantic relatedness of descriptions to the key words in the picture, were highest in the High MoCA group and had the greatest decline over 6-months in the MCI/AD group. **(D)** Syntactic complexity scores, reflecting the complexity of the sentences used, were highest in the High MoCA group.

To determine if speech aggregate scores were sensitive to differences between groups, we first tested the effect of group (High MoCA, Low MoCA, MCI/AD) on each aggregate score, controlling for visit (baseline, 6-months), sex, age, and years of education. Four of the eight aggregate scores had significant group differences: Word finding difficulty [*F*_(2, 44)_ = 10.03, *p* < 0.001], Information units [*F*_(2, 44)_ = 8.10, *p* < 0.001], Global coherence [*F*_(2, 44)_ = 4.11, *p* = 0.02], and Syntactic complexity [*F*_(2, 44)_ = 6.38, *p* = 0.004] (Figure 2). Follow-up pairwise testing using least square mean differences demonstrated significant differences between the High MoCA group and the MCI/AD group on all four scores (*p*'s < 0.01), and between the High MoCA group and Low MoCA group on Word finding difficulty (*p* < 0.001), Information units (*p* = 0.008), and Syntactic complexity (*p* = 0.008), but not Global coherence (*p* = 0.23).

**Figure 3:**
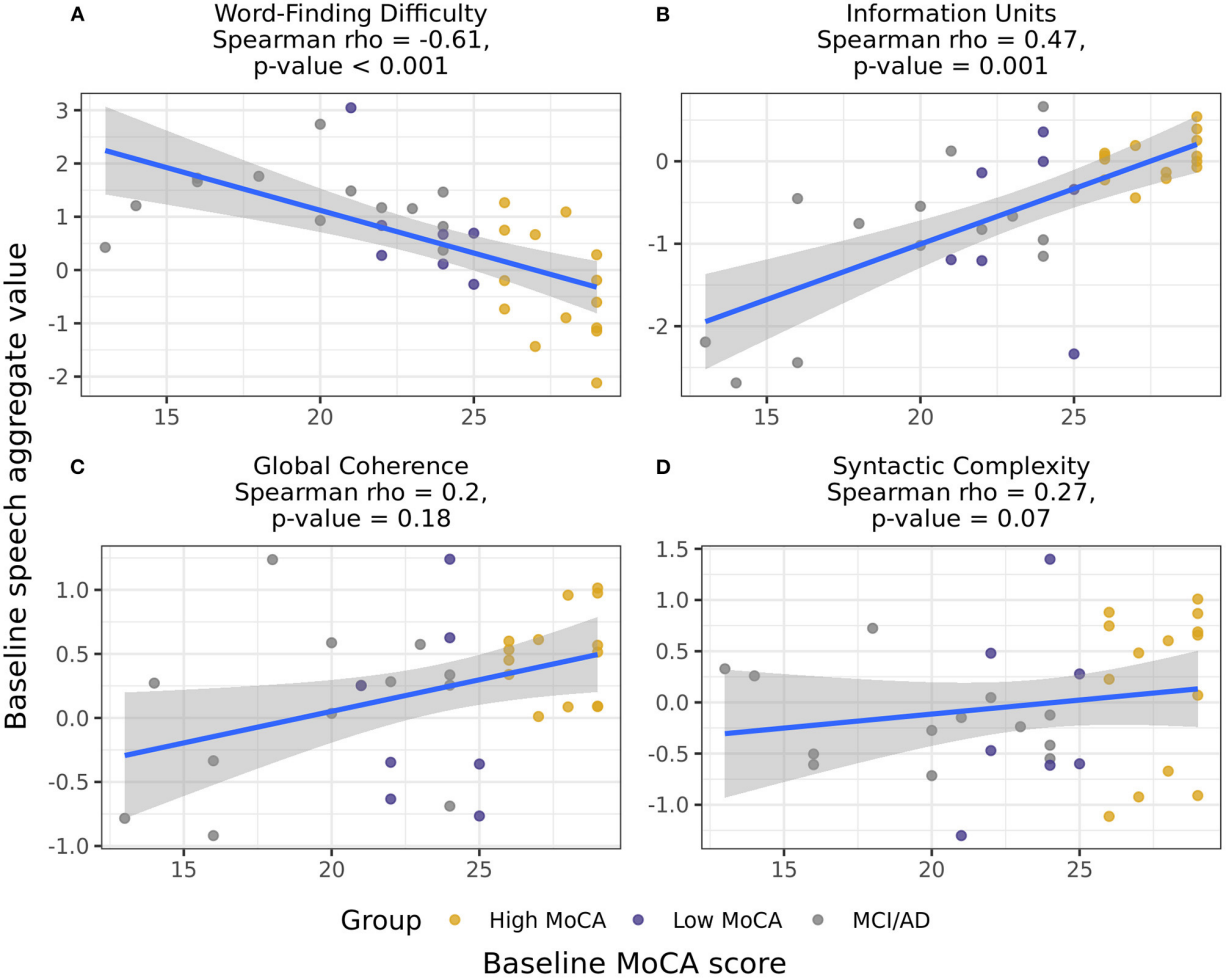
Correlations between the baseline speech aggregates scores and baseline MoCA scores, including participants in all three groups. Word-finding difficulty **(A)** and Information units **(B)** had significant correlations with baseline MoCA scores, indicating that those with higher MoCA scores demonstrated less word-finding difficulty and increased accurate information content in their descriptions. Global coherence **(C)** and Syntactic complexity **(D)** were not significantly correlated with baseline MoCA scores.

Combining groups and using Spearman partial correlations to test for continuous associations between baseline MoCA scores and baseline speech scores, we found that Word finding difficulty [*r*_(49)_ = −0.61, *p* < 0.001] and Information units [*r*_(49)_ = 0.47, *p* = 0.001] had moderate correlations with MoCA scores (Figure 3). The associations between MoCA scores and Global coherence [*r*_(49)_ = 0.20, *p* = 0.18] and Syntactic complexity [*r*_(49)_ = 0.27, *p* = 0.07] were positive but did not reach significance. Baseline MoCA scores were also significantly correlated with the change in Information units from baseline to 6-months [*r*_(49)_ = 0.39, *p* = 0.007], illustrating that those with lower MoCA scores at baseline had larger decreases in the informativeness of their descriptions over time.

### Change in Speech and Clinical Scores From Baseline to 6-Months Follow Up

For the aggregate scores that differed between groups, we next tested whether these scores showed differential rates of change between baseline and 6-month visits by testing for a group by visit interaction, again controlling for age, sex, and years of education. Global coherence was the only score to show a significant interaction [*F*_(2, 47)_ = 4.05, *p* = 0.02], reflecting a greater rate of decline in the MCI/AD group over 6-months than the High MoCA group (*p* = 0.007, Figure 2C).

As a comparison, we also examined the rates of change from baseline to 6-months in clinical scores. MoCA scores did not have a significant effect of visit [*F*_(1, 47)_ = 0.001, *p* = 0.97] or interaction between group and visit [*F*_(2, 44)_ = 0.59, *p* = 0.56]. ADAS-Cog scores also did not have significant effects of visit [*F*_(1, 32)_ = 2.23, *p* = 0.15], or a group by visit interaction [*F*_(2, 29)_ = 2.48, *p* = 0.10]. The MCI/AD group completed additional clinical measures, so visits were compared in this group only using linear models testing for the effect of visit, controlling for age, sex, and years of education. None of the scores on standard neuropsychological tests (SDMT, HVLT, JLO, digit span, Trails A and B) had significant change from baseline to 6-months (all *p*'s > 0.10).

### Test-Retest Reliability of Speech Scores

Finally, we tested the Pearson correlation between baseline and 1-month visits to determine the test-retest reliability of the aggregate scores over the 1-month period, in which we would expect little to no clinical change. Word finding difficulty [*r*_(46)_ = 0.83, *p* < 0.001] and Information units [*r*_(46)_ = 0.69, *p* < 0.001] had the highest associations between scores at baseline and 1-month, while Global coherence [*r*_(46)_ = 0.38, *p* = 0.007] and Syntactic complexity [*r*_(46)_ = 0.53, *p* < 0.001] had moderate, but still significant, correlations between the first two visits.

## Discussion

This proof-of-concept study showed that a digital speech assessment can be used to derive measures of linguistic abilities which are sensitive to early signs of cognitive impairment in older adults. Four language scores, relating to word-finding difficulty, syntactic complexity, information content, and coherence, were sensitive to detect differences between cognitively healthy older adults and those with MCI or early AD. All scores but the coherence score were additionally able to detect significant differences between community-recruited older adults with high vs. low scores on a widely used cognitive screening measure, indicating that these linguistic differences may reflect early cognitive changes that occur prior to a clinical diagnosis. The word-finding difficulty and information content scores were significantly correlated with MoCA scores, and had the highest test-retest reliability between baseline and 1-month testing sessions.

These findings are consistent with previous work suggesting that both the pacing and content of speech is affected in MCI and AD, and extends those findings to suggest that these differences may be detectable even prior to diagnosis. Several previous studies have reported that speech is slower and contains more pausing in MCI and AD (14, 47, 48, 51), consistent with the finding that the word finding difficulty score, which relates to speech rate and pauses, differed between groups. Content changes, resulting in less informative and coherent speech, are some of the most widely reported changes to occur in MCI and AD (14, 25, 42, 47, 49, 51, 57). The information unit and global coherence scores in the present study replicate these findings and add to the literature suggesting that reductions in linguistic content may be sensitive and early markers of cognitive impairment. The finding that syntactic complexity also differed between groups is consistent with some previous reports, which report shorter and simpler sentences in MCI and AD (14, 25, 47). While several previous studies have reported lexical changes in AD, often relating to increased pronoun use and use of more frequent words (14, 19, 25, 47), the lexical richness score did not have detectable differences between groups in the present study. It is difficult to make conclusions based on a null result, but this may suggest that the lexical richness score needs refining to detect vocabulary changes in MCI and AD.

When language scores were compared at 6-month follow-up, the global coherence score had a significant interaction between visit and group, demonstrating greater decline in the MCI/AD group compared to the High MoCA score group. Interestingly, combined with the group comparisons above, this suggests that the coherence of language may be affected later in the course of disease, but that in those with MCI or early AD, this measure declines more rapidly. Thus, this measure may be well-suited to sensitive measurement of change over time in those with MCI and AD. Notably, none of the neuropsychological tests, including the MoCA and the ADAS-Cog, showed significant change in the MCI/AD group over the same time period, further suggesting that the global coherence score may represent increased sensitivity over traditional clinical measures. This finding has potential implications for longitudinal measurement of symptoms, but requires replication in a larger sample and over a longer time period. The information unit score also showed trends of greater decline in the low MoCA and MCI/AD groups, which may be significant in larger samples or longer time periods.

This study demonstrates the utility of digital speech assessments for detecting and tracking cognitive impairment relating to MCI and AD. The picture description task, which was the focus of this paper, typically takes <5 min to administer and approximates spontaneous, natural speech. While the speech data in this study was collected in-person, speech has the potential to be collected remotely, and doesn't require the involvement of a clinician or any invasive medical procedures. Anecdotally, participants have reported enjoying this task and that it doesn't feel like they are taking a test, owing to its open-ended nature. Linguistic scores, automatically and objectively computed based on this task, demonstrated utility in differentiating participants based on diagnosis or the outcome of a commonly used cognitive screening tool. Longitudinally, these scores may have increased sensitivity to track disease progression compared to current clinical tools (58). Another advantage compared to classification models of speech in AD is that the scores generated in the present study are interpretable, reflecting different domains of the fluency, structure, and content of language. These scores provide more insight into the types of language changes that occur in MCI and AD, and how they may be differentially suited for early detection or longitudinal monitoring.

Limitations of this study include that the aggregate scores focused on linguistic features and did not include an acoustic feature aggregate. While previous studies have reported that acoustic features differ in AD (14, 18), the focus of this study was on linguistic features, since they are more clinically interpretable and in previous work have been shown to be sufficient for detection of MCI and AD (13, 19). Additionally, the samples were small, and the longitudinal follow up was limited to 6-months. Most clinical measures are able to detect progression of MCI and AD in periods of 1 year or longer (59), so the design of this study may have limited our ability to explore the longitudinal sensitivity of speech assessments. Future work is required to further explore the longitudinal profiles of language symptoms in MCI and AD. In addition, in this study we did not have access to other gold standard biomarkers of MCI and AD, including genetic markers, amyloid and tau concentrations or neuroimaging. In future work, comparing language patterns to these more established disease biomarkers will help further validate the disease-relevance of speech-based measures.

## Data Availability Statement

The raw data supporting the conclusions of this article will be made available by the authors, without undue reservation.

## Ethics Statement

The studies involving human participants were reviewed and approved by Advarra. The patients/participants provided their written informed consent to participate in this study.

## Author Contributions

WS and LK contributed to conception and design of the study. WS oversaw data collection. JR and MX performed the statistical analysis. JR wrote the first draft of the manuscript. All authors contributed to manuscript revision, read, and approved the submitted version.

## Funding

Data collection for this project was partially supported by a Neurotech Early Research & Development (NERD) project grant from the Ontario Brain Institute (OBI) and funding from the Ontario Centre of Innovation.

## Conflict of Interest

The authors of this article are employees of Winterlight Labs.

## Publisher's Note

All claims expressed in this article are solely those of the authors and do not necessarily represent those of their affiliated organizations, or those of the publisher, the editors and the reviewers. Any product that may be evaluated in this article, or claim that may be made by its manufacturer, is not guaranteed or endorsed by the publisher.
