# Supplementary material for: Using Digital Speech Assessments to Detect Early Signs of Cognitive Impairment
Source: Front Digit Health. 2021 Oct 27;3:749758. doi: 10.3389/fdgth.2021.749758 (PMC8579012; doi:10.3389/fdgth.2021.749758)
Supplement: Supplementary file 1 [file Table_1.DOCX]

Supplementary Table 1. Component variables of each speech aggregate score.

| **Speech aggregate** | **Speech variable name** | **Speech variable description** | **Weight** |
| --- | --- | --- | --- |
| Discourse mapping | ave_cos_dist | The sum of all cosine distances between each unique utterance pair divided by the total number of unique pairs. Repetition yields low value. | 1 |
| Discourse mapping | cos_cutoff_00 | Fraction of utterance pairs below the cosine similarity threshold of 0.001. The higher the fraction the more repetition. | -1 |
| Discourse mapping | cos_cutoff_03 | Fraction of utterance pairs below the cosine similarity threshold of 0.003. The higher the fraction the more repetition. | -1 |
| Discourse mapping | cos_cutoff_05 | Fraction of utterance pairs below the cosine similarity threshold of 0.005. The higher the fraction the more repetition. | -1 |
| Discourse mapping | graph_num_edges | Number of paths between sequential content words. | 1 |
| Discourse mapping | graph_num_nodes | Number of content words. | 1 |
| Discourse mapping | graph_self_loop_1 | Number of self-loop node paths. | -1 |
| Discourse mapping | min_cos_dist | The minimum cosine difference between an utterance pair divided by the number of pairs. Repetition yields low value. | 1 |
| Global coherence | global_coherence_GloVe_100_avg_dist | Average cosine distance between each utterance and its closest content unit centroid using the GloVe word vector model with 100-dimensional vectors. | -1 |
| Global coherence | global_coherence_GloVe_100_max_dist | Maximum cosine distance between each utterance and its closest content unit centroid using the GloVe word vector model with 100-dimensional vectors. | -1 |
| Global coherence | global_coherence_GloVe_100_min_dist | Minimum cosine distance between each utterance and its closest content unit centroid using the GloVe word vector model with 100-dimensional vectors. | -1 |
| Global coherence | global_coherence_GloVe_200_avg_dist | Average cosine distance between each utterance and its closest content unit centroid using the GloVe word vector model with 200-dimensional vectors. | -1 |
| Global coherence | global_coherence_GloVe_200_max_dist | Maximum cosine distance between each utterance and its closest content unit centroid using the GloVe word vector model with 200-dimensional vectors. | -1 |
| Global coherence | global_coherence_GloVe_200_min_dist | Minimum cosine distance between each utterance and its closest content unit centroid using the GloVe word vector model with 200-dimensional vectors. | -1 |
| Global coherence | global_coherence_GloVe_300_avg_dist | Average cosine distance between each utterance and its closest content unit centroid using the GloVe word vector model with 300-dimensional vectors. | -1 |
| Global coherence | global_coherence_GloVe_300_max_dist | Maximum cosine distance between each utterance and its closest content unit centroid using the GloVe word vector model with 300-dimensional vectors. | -1 |
| Global coherence | global_coherence_GloVe_300_min_dist | Minimum cosine distance between each utterance and its closest content unit centroid using the GloVe word vector model with 300-dimensional vectors. | -1 |
| Global coherence | global_coherence_GloVe_50_avg_dist | Average cosine distance between each utterance and its closest content unit centroid using the GloVe word vector model with 50-dimensional vectors. | -1 |
| Global coherence | global_coherence_GloVe_50_max_dist | Maximum cosine distance between each utterance and its closest content unit centroid using the GloVe word vector model with 50-dimensional vectors. | -1 |
| Global coherence | global_coherence_GloVe_50_min_dist | Minimum cosine distance between each utterance and its closest content unit centroid using the GloVe word vector model with 50-dimensional vectors. | -1 |
| Global coherence | global_coherence_Google_300_avg_dist | Average cosine distance between each utterance and its closest content unit centroid using the Google word vector model with 300-dimensional vectors. | -1 |
| Global coherence | global_coherence_Google_300_max_dist | Maximum cosine distance between each utterance and its closest content unit centroid using the Google word vector model with 300-dimensional vectors. | -1 |
| Global coherence | global_coherence_Google_300_min_dist | Minimum cosine distance between each utterance and its closest content unit centroid using the Google word vector model with 300-dimensional vectors. | -1 |
| Information units | info_units_bool_count | Number of distinct content units. | 1 |
| Information units | info_units_bool_count_action | Number of distinct action word content units. | 1 |
| Information units | info_units_bool_count_location | Number of distinct location word content units. | 1 |
| Information units | info_units_bool_count_object | Number of distinct object word content units. | 1 |
| Information units | info_units_bool_count_subject | Number of distinct subject content units. | 1 |
| Information units | info_units_count | Number of content units. | 1 |
| Information units | info_units_count_action | Number of action word content units. | 1 |
| Information units | info_units_count_location | Number of location word content units. | 1 |
| Information units | info_units_count_object | Number of object word content units. | 1 |
| Information units | info_units_count_subject | Number of subject content units. | 1 |
| Lexical richness | age_of_acquisition | Age at which each word was learned (according to lexical norm literature) averaged over all words in transcript. | 1 |
| Lexical richness | avg_word_length | Average number of characters per word. | 1 |
| Lexical richness | brunet | Quantification of lexical richness by multiplying total text length by the total number of unique words, multiplied by a scaling constant. | -1 |
| Lexical richness | category_demonstratives | Count of demonstratives. | -1 |
| Lexical richness | category_function | Count of function words. | -1 |
| Lexical richness | category_inflected_verbs | Count of inflected verbs. | 1 |
| Lexical richness | category_light_verbs | Count of light verbs. | -1 |
| Lexical richness | category_subordinate | Count of subordinating conjunctions. | 1 |
| Lexical richness | familiarity | Familiarity of each word (according to lexical norm literature) averaged over all words in transcript. | -1 |
| Lexical richness | frequency | Frequency of each word (according to lexical norm literature) averaged over all words in transcript. | -1 |
| Lexical richness | imageability | Imageability of each word (according to lexical norm literature) averaged over all words in transcript. | -1 |
| Lexical richness | MATTR_10 | Moving average ratio of total number of unique words (type) total number of words (tokens) within window of 10 words. | 1 |
| Lexical richness | MATTR_20 | Moving average ratio of total number of unique words (type) total number of words (tokens) within window of 20 words. | 1 |
| Lexical richness | MATTR_30 | Moving average ratio of total number of unique words (type) total number of words (tokens) within window of 30 words. | 1 |
| Lexical richness | MATTR_40 | Moving average ratio of total number of unique words (type) total number of words (tokens) within window of 40 words. | 1 |
| Lexical richness | MATTR_50 | Moving average ratio of total number of unique words (type) total number of words (tokens) within window of 50 words. | 1 |
| Lexical richness | NOUN_age_of_acquisition | Age at which each noun was learned (according to lexical norm literature) averaged over all nouns in transcript. | 1 |
| Lexical richness | NOUN_familiarity | Familiarity of each noun (according to lexical norm literature) averaged over all nouns in transcript. | -1 |
| Lexical richness | NOUN_frequency | Frequency of each noun (according to lexical norm literature) averaged over all nouns in transcript. | -1 |
| Lexical richness | NOUN_imageability | Imageability of each noun (according to lexical norm literature) averaged over all nouns in transcript. | -1 |
| Lexical richness | noun_ratio | Ratio of nouns to the sum of nouns and verbs. | 1 |
| Lexical richness | pos_ADJ | Count of adjectives. | 1 |
| Lexical richness | pos_ADP | Count of adpositions. | 1 |
| Lexical richness | pos_ADV | Count of adverbs. | 1 |
| Lexical richness | pos_CCONJ | Count of coordination conjunctions. | 1 |
| Lexical richness | pos_INTJ | Count of interjections. | -1 |
| Lexical richness | pos_NOUN | Count of nouns. | 1 |
| Lexical richness | pos_PRON | Count of pronouns. | -1 |
| Lexical richness | pos_PROPN | Count of proper nouns. | 1 |
| Lexical richness | pos_VERB | Count of verbs. | 1 |
| Lexical richness | propositional_density_ratio_propositions | The average number of propositions/ideas expressed per 10 words as measured by elementary propositions: verbs, adverbs, adjectives, conjunctions, and prepositions. | 1 |
| Lexical richness | prp_ratio | Ratio of pronouns to the sum of pronouns and nouns. | -1 |
| Lexical richness | tag_IN | Count of prepositions and subordinating conjunctions. | 1 |
| Lexical richness | tag_JJ | Count of adjectives. | 1 |
| Lexical richness | tag_JJR | Count of comparative adjectives. | 1 |
| Lexical richness | tag_JJS | Count of superlative adjectives. | 1 |
| Lexical richness | tag_MD | Count of modal verbs. | 1 |
| Lexical richness | tag_NN | Count of singular nouns. | 1 |
| Lexical richness | tag_NNP | Count of plural nouns. | 1 |
| Lexical richness | tag_NNS | Count of singular proper nouns. | 1 |
| Lexical richness | tag_PRP | Count of personal pronouns. | -1 |
| Lexical richness | tag_RB | Count of adverbs. | 1 |
| Lexical richness | tag_RBR | Count of comparative adverbs. | 1 |
| Lexical richness | tag_UH | Count of interjections. | -1 |
| Lexical richness | tag_VB | Count of verbs, base form. | 1 |
| Lexical richness | tag_VBD | Count of verbs, past tense. | 1 |
| Lexical richness | tag_VBG | Count of verbs, gerund. | 1 |
| Lexical richness | tag_VBN | Count of verbs, past participle. | 1 |
| Lexical richness | tag_VBP | Count of verbs, non-3^rd^ person singular present. | 1 |
| Lexical richness | tag_VBZ | Count of verbs, 3^rd^ person singular present. | 1 |
| Lexical richness | TTR | Ratio of unique to total number of words. | 1 |
| Lexical richness | VERB_age_of_acquisition | Age at which each verb was learned (according to lexical norm literature) averaged over all verbs in transcript. | 1 |
| Lexical richness | VERB_familiarity | Familiarity of each verb (according to lexical norm literature) averaged over all verbs in transcript. | -1 |
| Lexical richness | VERB_frequency | Frequency of each verb (according to lexical norm literature) averaged over all verbs in transcript. | -1 |
| Lexical richness | VERB_imageability | Imageability of each verb (according to lexical norm literature) averaged over all verbs in transcript. | -1 |
| Local coherence | local_coherence_GloVe_100_avg_dist | Average cosine distance between successive utterances using the GloVe word vector model with 100-dimensional vectors. | -1 |
| Local coherence | local_coherence_GloVe_100_max_dist | Maximum cosine distance between successive utterances using the GloVe word vector model with 100-dimensional vectors. | -1 |
| Local coherence | local_coherence_GloVe_100_min_dist | Minimum cosine distance between successive utterances using the GloVe word vector model with 100-dimensional vectors. | -1 |
| Local coherence | local_coherence_GloVe_200_avg_dist | Average cosine distance between successive utterances using the GloVe word vector model with 200-dimensional vectors. | -1 |
| Local coherence | local_coherence_GloVe_200_max_dist | Maximum cosine distance between successive utterances using the GloVe word vector model with 200-dimensional vectors. | -1 |
| Local coherence | local_coherence_GloVe_200_min_dist | Minimum cosine distance between successive utterances using the GloVe word vector model with 200-dimensional vectors. | -1 |
| Local coherence | local_coherence_GloVe_300_avg_dist | Average cosine distance between successive utterances using the GloVe word vector model with 300-dimensional vectors. | -1 |
| Local coherence | local_coherence_GloVe_300_max_dist | Maximum cosine distance between successive utterances using the GloVe word vector model with 300-dimensional vectors. | -1 |
| Local coherence | local_coherence_GloVe_300_min_dist | Minimum cosine distance between successive utterances using the GloVe word vector model with 300-dimensional vectors. | -1 |
| Local coherence | local_coherence_GloVe_50_avg_dist | Average cosine distance between successive utterances using the GloVe word vector model with 50-dimensional vectors. | -1 |
| Local coherence | local_coherence_GloVe_50_max_dist | Maximum cosine distance between successive utterances using the GloVe word vector model with 50-dimensional vectors. | -1 |
| Local coherence | local_coherence_GloVe_50_min_dist | Minimum cosine distance between successive utterances using the GloVe word vector model with 50-dimensional vectors. | -1 |
| Local coherence | local_coherence_Google_300_avg_dist | Average cosine distance between successive utterances using the Google word vector model with 300-dimensional vectors. | -1 |
| Local coherence | local_coherence_Google_300_max_dist | Maximum cosine distance between successive utterances using the Google word vector model with 300-dimensional vectors. | -1 |
| Local coherence | local_coherence_Google_300_min_dist | Minimum cosine distance between successive utterances using the Google word vector model with 300-dimensional vectors. | -1 |
| Sentiment | NOUN_sentiment_valence | Average valence score (according to the lexical norm literature) of each noun, averaged over all nouns in the transcript. | 1 |
| Sentiment | sentiment_valence | Average valence score (according to the lexical norm literature) of each word, averaged over all words in the transcript. | 1 |
| Sentiment | VERB_sentiment_valence | Average valence score (according to the lexical norm literature) of each verb, averaged over all verbs in the transcript. | 1 |
| Syntactic complexity | ADJP_->_JJ | Adjective phrase consisting of an adjective. Fraction of the number of times this production occurs, over the number of all syntactic productions found in transcript. | 1 |
| Syntactic complexity | ADVP_->_RB | Adverb phrase consisting of an adverb. Fraction of the number of times this production occurs, over the number of all syntactic productions found in transcript. | 1 |
| Syntactic complexity | constituency_average_NP_length | Average word length of noun phrases. | 1 |
| Syntactic complexity | constituency_average_PP_length | Average word length of prepositional phrases. | 1 |
| Syntactic complexity | constituency_average_VP_length | Average word length of verb phrases. | 1 |
| Syntactic complexity | constituency_avg_depth | Average Yngve depth of utterances. | 1 |
| Syntactic complexity | constituency_max_depth | Maximum Yngve depth of utterances. | 1 |
| Syntactic complexity | constituency_NP_type_prop | Proportion of words that belong to noun phrases. | 1 |
| Syntactic complexity | constituency_NP_type_rate | Number of noun phrases divided by total words. | 1 |
| Syntactic complexity | constituency_PP_type_prop | Proportion of words that belong to prepositional phrases. | 1 |
| Syntactic complexity | constituency_PP_type_rate | Number of prepositional phrases divided by total words. | 1 |
| Syntactic complexity | constituency_total_depth | Total Yngve depth of utterances. | 1 |
| Syntactic complexity | constituency_treeheight | Average utterance parse tree height. | 1 |
| Syntactic complexity | constituency_VP_type_prop | Proportion of words that belong to verb phrases. | 1 |
| Syntactic complexity | constituency_VP_type_rate | Number of verb phrases divided by total words. | 1 |
| Syntactic complexity | INTJ_->_UH | Interjections. Fraction of the number of times this production occurs, over the number of all syntactic productions found in transcript. | -1 |
| Syntactic complexity | Lu_C/S | Number of clauses per sentence. | 1 |
| Syntactic complexity | Lu_C/T | Number of clauses per t-unit. | 1 |
| Syntactic complexity | Lu_CN/C | Number of complex nominals per clause. | 1 |
| Syntactic complexity | Lu_CN/T | Number of complex nominals per t-unit. | 1 |
| Syntactic complexity | Lu_CP | Number of coordinate phrases. | 1 |
| Syntactic complexity | Lu_CP/C | Number of coordinate phrases per clause. | 1 |
| Syntactic complexity | Lu_CP/T | Number of coordinate phrases per t-unit. | 1 |
| Syntactic complexity | Lu_CT | Number of complex t-units. | 1 |
| Syntactic complexity | Lu_CT/T | Number of complex t-units per t-unit. | 1 |
| Syntactic complexity | Lu_DC | Number of dependent clauses. | 1 |
| Syntactic complexity | Lu_DC/C | Number of dependent clauses per clause. | 1 |
| Syntactic complexity | Lu_DC/T | Number of dependent clauses per t-unit. | 1 |
| Syntactic complexity | Lu_MLC | Mean length of clause. | 1 |
| Syntactic complexity | Lu_MLS | Mean length of sentence. | 1 |
| Syntactic complexity | Lu_MLT | Mean length of t-unit. | 1 |
| Syntactic complexity | Lu_T | Number of t-units. | 1 |
| Syntactic complexity | Lu_T/S | Number of t-units per sentence. | 1 |
| Syntactic complexity | Lu_VP | Number of verb phrases. | 1 |
| Syntactic complexity | Lu_VP/T | Number of verb phrases per t-unit. | 1 |
| Syntactic complexity | max_utt_len | Maximum utterance length. | 1 |
| Syntactic complexity | min_utt_len | Minimum utterance length. | 1 |
| Syntactic complexity | MLU | Mean utterance length. | 1 |
| Syntactic complexity | NP_->_DT_JJ_NN | Noun phrase consisting of a determiner, an adjective and a noun. Fraction of the number of times this production occurs, over the number of all syntactic productions found in transcript. | 1 |
| Syntactic complexity | NP_->_DT_JJ_NNS | Noun phrase consisting of a determiner, an adjective and a plural noun. Fraction of the number of times this production occurs, over the number of all syntactic productions found in transcript. | 1 |
| Syntactic complexity | NP_->_DT_NN | Noun phrase consisting of a determiner and a noun. Fraction of the number of times this production occurs, over the number of all syntactic productions found in transcript. | 1 |
| Syntactic complexity | NP_->_DT_NN_NN | Noun phrase consisting of a determiner and two nouns. Fraction of the number of times this production occurs, over the number of all syntactic productions found in transcript. | 1 |
| Syntactic complexity | NP_->_DT_NNS | Noun phrase consisting of a determiner and a plural noun. Fraction of the number of times this production occurs, over the number of all syntactic productions found in transcript. | 1 |
| Syntactic complexity | NP_->_NN | Noun phrase consisting of a noun. Fraction of the number of times this production occurs, over the number of all syntactic productions found in transcript. | 1 |
| Syntactic complexity | NP_->_NN_NN | Noun phrase consisting of two nouns. Fraction of the number of times this production occurs, over the number of all syntactic productions found in transcript. | 1 |
| Syntactic complexity | NP_->_NNS | Noun phrase consisting of a plural noun. Fraction of the number of times this production occurs, over the number of all syntactic productions found in transcript. | 1 |
| Syntactic complexity | PP_->_IN | Prepositional phrase consisting of a preposition. Fraction of the number of times this production occurs, over the number of all syntactic productions found in transcript. | 1 |
| Syntactic complexity | ROOT_->_FRAG | Fragment. Fraction of the number of times this production occurs, over the number of all syntactic productions found in transcript. | -1 |
| Word finding difficulty | avg_word_duration | Average word duration. | 1 |
| Word finding difficulty | filled_pauses | Filled pause count. | 1 |
| Word finding difficulty | hesitation | Proportion of utterances beginning with a pause. | 1 |
| Word finding difficulty | long_pause_count_normalized | Number of unfilled pauses longer than 2s divided by the length of audio. | 1 |
| Word finding difficulty | mean_pause_duration | Average pause duration. | 1 |
| Word finding difficulty | medium_pause_count_normalized | Number of unfilled pauses 1-2s long divided by the length of audio. | 1 |
| Word finding difficulty | NID | Number of words that are unidentifiable or not in the dictionary. | 1 |
| Word finding difficulty | pause_word_ratio | Ratio of unvoiced to voiced segments. | 1 |
| Word finding difficulty | short_pause_count_normalized | Number of unfilled pauses <1s long divided by the length of audio. | 1 |
| Word finding difficulty | speech_rate | Number of words per minute. | -1 |
| Word finding difficulty | uh | Count of “uh” filled pauses. | 1 |
| Word finding difficulty | um | Count of “um” filled pauses. | 1 |
| Word finding difficulty | unfilled_pauses | Unfilled pause count. | 1 |
